# The relationship between perceived executive function and self-reported self-management behaviour in adults with type 1 diabetes

**DOI:** 10.1177/13591053251341787

**Published:** 2025-06-25

**Authors:** Lynne Shanley, Daniel Powell, Julia Allan

**Affiliations:** 1 University of Aberdeen, UK; 2 University of Stirling, UK

**Keywords:** adults, chronic illness, diabetes, executive function, self-management

## Abstract

This mixed-method study examined whether and how perceived executive function (EF) is linked to self-reported self-management in 173 people diagnosed with Type 1 diabetes (T1D) during adulthood, combining a cross-sectional survey with thematic analysis of 11 interviews. Stronger global EF significantly predicted better self-management (*B* = −0.04, *t*(165) = 4.15; *p* < 0.001) after controlling for demographic factors. Stronger perceptions of EF correlated with better self-reported adherence to dietary behaviour, glucose monitoring and physical activity, but not medication-taking or cooperation with healthcare teams. Qualitative interviews identified key challenges in self-management requiring stronger EF including planning behaviours, maintaining attention and vigilance over time and responding flexibly to changing demands. Strategies which reduce demands on EF, such as establishing routines and delegating control of tasks, helped to improve self-management. Adults with perceived EF impairments may struggle to effectively manage T1D, suggesting supportive interventions should aim to reduce the cognitive demands of self-management.

## Introduction

T1D affects approximately 8.4 million people worldwide with prevalence rates projected to double in the next two decades (Mahase, 2022). Its physical and mental consequences can be severe, increasing the risk of cardiovascular disease, stroke, sight loss, kidney failure, amputation and premature death (Wei et al., 2022), and psychiatric illnesses including mood, eating and anxiety disorders (Collins et al., 2009; de Groot et al., 2016). There is no cure for T1D but engagement in co-ordinated self-management behaviours may help control symptoms and delay the onset of complications. Key self-management behaviours include healthy eating, exercise and engaging with the health care team. They also include taking medication, and insulin therapy to regulate blood glucose, administered either through multiple daily injections or insulin pumps which deliver a continuous supply of insulin. Monitoring blood glucose is similarly critical and typically uses either traditional finger-prick testing or continuous glucose monitoring (CGM) systems. CGMs offer the advantage of real-time readings that may reduce the burden of self-monitoring, but access and affordability remain significant barriers to widespread use. Consistent engagement in self-management correlates with improved quality of life (Saleh et al., 2014), lower depression (Gonzalez et al., 2008), reduced vascular complications (Chen et al., 2015) and decreased healthcare expenditure (Fukuda and Mizobe, 2017). As such, international healthcare guidelines recommend self-management for optimal T1D outcomes (American Association of Diabetes Educators, 2020; Amiel et al., 2015).

The effective execution and coordination of self-management behaviours in T1D is complex, requiring sustained effort in monitoring, vigilance, memory and planning. Perhaps unsurprisingly, global adherence to diabetes self-management is frequently suboptimal (Peyrot et al., 2005). Moreover, current self-management interventions aimed at improving adherence in diabetes show limited effectiveness (Wong et al., 2020). These findings underscore the need to identify factors affecting self-management engagement to develop more effective behaviour change interventions for T1D care.

One factor that has received attention for its role in facilitating effective T1D self-management is executive function (EF). EF can be viewed as a collection of top-down, higher-level mental processes involved in controlling goal-directed thoughts and actions, including the capacity for planning, reasoning and problem-solving (Diamond, 2013). It is commonly agreed that EF comprises three components; inhibition, working memory (*updating*) and cognitive flexibility (*shifting)* unified by a single mechanism (Diamond, 2013; Miyake et al., 2000). Inhibition refers to the ability to intentionally control and suppress thoughts and activities, in turn supporting self-control and discipline, focussed attention and urge suppression. Working memory, operating in tandem with inhibition, permits relevant information to be held in mind and updated such that current events may be interpreted, and new ideas incorporated. Cognitive flexibility refers to the ability to flexibly shift focus between tasks, goals and processes enabling the consideration of different perspectives and the simultaneous solution of multiple problems (Diamond, 2013).

As EF underpins goal-directed and self-regulatory tasks, EF deficits may impair management of specific health-related behaviours. EF appears to be impaired in individuals with T1D (Broadley, White and Andrew, 2017) and a systematic review by Ding et al. (2021) indicates a significant association between EF weaknesses, as reported by individuals or caregivers, and lower self-management in adolescents and young adults with T1D. This is particularly significant given that T1D is often diagnosed during adolescence, and that early glycaemic control offers long-term health benefits (Diabetes Control and Complications Trial Research Group, 1993). However, nearly 50% of new T1D cases arise during adulthood (Thomas et al., 2018) where adherence may be further challenged by firmly established habits and routines. To the authors’ knowledge, no studies have examined the relationship between EF and self-management in people diagnosed with T1D during adulthood nor have they gone beyond quantitative studies of global EF to explore the relative importance of specific facets of EF (e.g. working memory, inhibition) for distinct self-management behaviours (e.g. glucose monitoring, healthy eating), or the subjective role of EF in people’s lived experience of T1D management.

The current study aimed to address this literature gap by (i) using validated measures to quantitatively explore the link between perceived EF strength and self-reported self-management in people diagnosed with T1D during adulthood, (ii) determining the relative importance of different sub-facets of EF for different self-management behaviours in T1D and (iii) qualitatively exploring the role of EF in perceived difficulties with, and strategies used to facilitate better self-management.

## Methods

### Study design

This study was a mixed-method design. A cross-sectional online self-report questionnaire quantitatively investigated the relationship between EF strength and self-management adherence using validated measures. Semi-structured interviews qualitatively explored perceived EF-related self-management difficulties, and strategies used to aid T1D self-management.

### Ethical approval

This study was approved by The University of Aberdeen’s School of Medicine, Medical Sciences & Nutrition Research Ethics Board (SERB/654603). All participants gave informed consent before participation. Interview participants received a £15 Amazon voucher as reimbursement for their time.

### Quantitative study

#### Participants and recruitment

Eligible participants were adults who had a self-reported T1D diagnosis, diagnosed at least 6 months ago, and were 18 years or older at diagnosis. All participants were UK residents and able to speak the English language. Participants were recruited opportunistically through diabetes-specific social media pages (Facebook, Instagram, Twitter), online diabetes forums and through the online platform, Prolific.

Prior research indicates medium-to-large associations between subjective measures of EF and self-management in adolescents with T1D (Ding et al., 2021). An a priori sample size calculation in G*Power software estimated that a minimum sample size of 123 participants would be necessary to detect a medium-sized effect in regression analyses with 6 predictors, with 90% power and α = 0.05. To maximize the study’s statistical power, recruitment of participants continued until a prespecified study recruitment end date (September 31st, 2023).

#### Measures

##### Outcome variable: Diabetes self-management

The Diabetes Self-Management Questionnaire-Revised (DSMQ-R; Schmitt, 2022) assesses diabetes self-management incorporating recent innovations in glucose monitoring and updated diabetes-related language. The 20-item measure uses a 4-point Likert scale (0–3), with higher scores reflecting better self-management. Two items contribute only to total score, while 18 assess behaviour across five subscales: eating behaviour (6), medication taking (2), glucose monitoring (3), physical activity (3) and cooperation with diabetes team (4). The DSMQ-R demonstrates high internal consistency and adequate test-retest reliability over 3–6 months, both for the total scale and its subscales. It also demonstrates strong construct and convergent validity, with higher scores correlating with better glycaemic control (HbA1c) and other diabetes management measures, such as the Summary of Diabetes Self-Care Activities (Schmitt et al., 2022). Total and subscale scores were transformed to a scale range of 0–10 as recommended by the measure’s authors.

##### Predictor variable: Perceived global EF strength

The validated Behavioural Rating Inventory of Executive Function Scale for adults (BRIEF-A; Roth et al., 2005) assessed perceived EF strength. This 75-item self-report questionnaire, designed for adults 18–90 years of age, measures nine theoretically nonoverlapping EF sub-facets; inhibit, self-monitor, plan/organize, shift, initiate, task-monitoring, emotional control, working memory and organizing of materials. Respondents rate how often they experienced problems with behaviours linked to these facets in the past month on a 3-point Likert scale (1 = never; 2 = sometimes; 3 = often). The sum of raw scores provides a unitary measure of EF strength (‘Global Executive Composite’ or GEC). Higher scores indicate greater EF dysfunction. Raw scores for GEC, and the EF facets, were transformed into age-adjusted scores (Mean = 50, SD = 10; Roth et al., 2005). BRIEF-A demonstrates strong test-retest reliability over 4 weeks, high internal consistency and adequate construct validity correlating significantly with other EF measures including the Dysexecutive Questionnaire (Roth et al., 2005).

##### Demographic information

Participants self-reported gender, age, level of education, age at diagnosis and method of insulin control (self-administered vs automated).

##### Procedure

The online survey was completed in Qualtrics (www.qualtrics.com). After providing informed consent, participants were invited to complete the demographic items questionnaire, the BRIEF-A scale and the DSMQ-R scale. Participants were prompted to answer missed questions but could continue without answering if they wished. After completing the survey, participants were given debrief information and asked if they would be interested in taking part in the second study involving an online interview conducted over Microsoft Teams. Interested participants were asked to leave their email addresses for further information, and an invitation to participate.

##### Data analysis

There was no missing demographic or DSMQ-R data, however across 6 participants, 11 responses (0.08%) were omitted on the BRIEF-A measure. In line with the BRIEF-A manual, omitted items were assigned a score of 1. Multiple regression was used to predict self-management adherence from EF strength after controlling for age, years since diagnosis, gender, educational attainment and insulin delivery method. Pearson correlations were used to explore the pattern of associations between specific facets of EF and different self-management behaviours. As a sensitivity analysis, analyses were re-run excluding participants who scored above the recommended cut-offs on any of the BRIEF-A validity scales. IBM Statistical Package for Social Sciences (SPSS) for Windows version 26 was used for statistical analyses.

### Qualitative study

#### Participants

Participants for the qualitative study were selected through convenience sampling from those involved in Study 1. Twenty interested participants were invited for interviews; 12 provided written consent and 1 withdrew, leaving a final sample of 11 participants. This sample size aligns with recommendations for thematic analysis (Braun et al., 2016).

#### Semi-structured interview

Semi-structured interviews were used to capture candid, detailed narratives of the lived experience of managing T1D and were conducted and recorded virtually over Microsoft Teams. An interview topic guide was developed to explore participants’ feelings and behaviours relating to T1D self-management and to elicit information about perceived challenges and coping strategies. Conversation was focussed on the experience of diagnosis and living with T1D; daily life with T1D; challenges associated with living with T1D; changes noticed over time; and approaches and strategies used to live with T1D. Participants answered questions freely without constraint and were not obliged to answer any questions they did not wish to.

#### Data analysis

Interviews were transcribed verbatim. Transcripts were manually cross-checked with the recordings before being imported into NVIVO for coding. The analysis followed the six steps of thematic analysis as outlined by Braun and Clarke (2006) and Braun et al. (2016). Statements were coded and codes were grouped by a single researcher (LS) into recurring themes based on shared meaning. Themes were discussed with a second researcher (JA) before being finalized.

## Results

### Quantitative survey results

One hundred and seventy-five participants completed the survey. Two participants did not provide their age and were excluded as accurate age-adjusted EF scores could not be computed, leaving 173 participants: *n* = 116 from online diabetes groups and forums, *n* = 57 from Prolific. A descriptive summary of the participant characteristics and scores on study variables is shown in Table 1.

**Table 1.** Participant characteristics.

	Mean (standard deviation)	Range (years)	Count (%)
*N*			173 (100%)
Age (years)	45.14 (12.20)	19–77	
Time since diagnosis (years)	13.91 (11.65)	1–52	
Gender			
Male			37 (21.40)
Female			135 (78.00)
Nonbinary/genderqueer/agender/gender fluid			1 (0.60)
Highest level of educational attainment			
Primary/elementary school			0 (0)
Secondary/high school			32 (18.50)
College/university			82 (47.40)
Postgraduate study			57 (32.90)
Prefer not to say			2 (1.20)
Insulin delivery method			
Calculate/inject insulin myself			117 (67.60)
Insulin pump/closed loop			56 (32.40)
			*N* (%) scoring >65 indicating dysfunction
EF strength (tGEC score)	58.95 (12.99)		52 (29.71)
Initiate	58.88 (12.71)		58 (33.52)
Inhibit	53.55 (11.15)		26 (15.03)
Shift	58.58 (11.54)		48 (27.75)
Working memory	61.10 (14.23)		61 (35.26)
Emotional control	59.51 (12.73)		53 (30.63)
Self-monitor	51.55 (11.75)		22 (12.72)
Plan/organize	56.42 (12.81)		47 (27.17)
Task monitor	57.66 (12.31)		48 (27.75)
Organize materials	54.73 (12.68)		38 (21.97)
DSMQ-R total score	6.52 (1.65)		
Eating behaviour	5.86 (1.91)		
Taking medication	7.71 (2.58)		
Glucose monitoring	7.07 (2.46)		
Physical activity	6.08 (2.64)		
Cooperation with diabetes team	6.65 (2.24)		

EF: executive function; tGEC: age-adjusted global executive composite; DSMQ-R: diabetes self-management questionnaire-revised.

T-scores for overall EF strength (tGEC) above 65 are typically considered to be indicative of potential EF impairments (Roth et al., 2005). The mean T-scores for overall EF strength and specific sub-facets ranged from 51.55 to 61.10, which is within the normal range of EF functioning. All participants scored within the acceptable range for the negativity and infrequency validity scales built into the BRIEF-A but three participants scored in the elevated range (≥8) for the inconsistency of response validity scale. Sensitivity analyses excluding these participants showed no substantial differences to results, so all are included in the results outlined below.

Multiple regression was used to predict self-management (DSMQ-R score) from EF strength (BRIEF-A score) after controlling for demographic variables. All assumptions of multiple regression were met. The model was significant and explained 24% of the variance in self-management (Adjusted *R*^2^ = 0.235, *F*(7, 165) = 8.547, *p* < 0.001). EF strength significantly and negatively predicted DSMQ-R score indicating that participants reporting poorer EF/greater dysfunction (higher tGEC score), tended to report poorer diabetes self-management (lower DSMQ-R score). As shown in Table 2, for every one unit increase in tGEC score there was a 0.037 unit decrease in DSMQ-R score (*B* = −0.037, *t*(165) = 4.147; *p* < 0.001). Relative to self-injecting methods of insulin delivery, the use of an insulin pump was associated with a significantly higher DSMQ-R score, that is, with better self-management (*B* = 0.791, *t*(165) = 3.212; *p* = 0.002). Age also significantly and positively predicted DSMQ-R score, indicating better self-management in older participants (*B* = 0.048, *t*(165) = 4.079; *p* < 0.001). Time since diagnosis, gender and education level did not significantly predict DSMQ-R scores.

**Table 2.** Multiple regression analysis predicting DSMQ-R score.

	Unstandardized coefficients		Standardized coefficients	*t*	Sig	95% confidence interval for B	
	*B*	Std. error	Beta			Lower bound	Upper bound
Constant	5.235	0.948		5.520	<0.001	3.362	7.107
Age	0.048	0.012	0.355	4.079	<0.001	0.025	0.071
Duration of diagnosis	−0.020	0.012	−0.143	−1.648	0.101	−0.045	0.004
Highest level of education	0.188	0.153	0.083	1.225	0.222	−0.115	0.491
Gender = male	−0.340	0.276	−0.085	−1.231	0.220	−0.055	−0.019
Gender = non-binary	0.657	1.466	0.030	0.488	0.655	−2.237	3.551
Insulin delivery method = I use an insulin pump	0.791	0.246	0.225	3.212	0.002	0.305	1.278
Global executive composite (tGEC)	−0.037	0.009	−0.290	−4.147	<0.001	−0.055	−0.019

Pearson correlations were used to quantify the association between total EF strength and adherence to different self-management behaviours, and between sub-facets of EF and self-management behaviour. As illustrated in Table 3, total EF strength was significantly and negatively associated with eating behaviour (*r* = −0.278, *p* < 0.001), glucose monitoring (*r* = −0.161, *p* < 0.035) and physical activity (*r* = −0.391, *p* < 0.001) indicating that greater EF dysfunction (higher tGEC scores) is associated with poorer management of these behaviours. However, total EF was not significantly associated with medication taking (*r* = 0.076, *p* = 0.323) or cooperation with the healthcare team (*r* = −0.116, *p* = 0.129). All EF sub-facets were significantly and negatively associated with self-management overall and with eating behaviour and physical activity (*r* values ranging from −0.160 to −0.416, *p* < 0.001), with the strongest association being between ability to initiate and physical activity (*r* = −0.416, *p* < 0.001). Glucose monitoring was associated with ability to inhibit (*r* = −0.156, *p* = 0.002), self-monitor (*r* = −0.159, *p* = 0.036) and plan/organize (*r* = −0.176, *p* = 0.002). Neither medication taking nor cooperation with the healthcare team were associated with any specific facets of EF.

**Table 3.** Pearson correlation coefficients (*r*) between different EF measures and self-management behaviours in adults with T1D.

	Eating behaviour	Medication taking	Glucose monitoring	Physical activity	Co-operation with health care team	Total score
Initiate	−0.238**	−0.080	−0.149	−0.416***	−0.075	−0.279***
Inhibit	−0.277***	−0.143	−0.156*	−0.237**	−0.146	−0.298***
Shift	−0.160*	−0.025	−0.056	−0.245**	−0.140	−0.196**
Working memory	−0.242**	−0.060	−0.104	−0.329***	−0.069	−0.255***
Emotional control	−0.181*	−0.004	−0.109	−0.251***	−0.129	−0.232**
Self-monitor	−0.232**	−0.091	−0.159*	−0.250***	−0.137	−0.279***
Plan/organize	−0.205**	−0.086	−0.175*	−0.360***	−0.095	−0.270***
Task monitor	−0.209**	−0.035	−0.123	−0.326***	−0.001	−0.219**
Organize materials	−0.207**	−0.008	−0.062	−0.304***	−0.022	−0.206**
GEC	−0.278***	−0.076	−0.161*	−0.391***	−0.116	−0.324***

GEC: global executive composite; DSMQ-R: diabetes self-management questionnaire-revised.

* *p* < 0.05. **p < 0.01. ***p < 0.001.

### Qualitative data analysis

Participant characteristics of those interviewed are displayed in Table 4. The mean age of the sample was 49.45 years (SD = 11.51), and a majority (8) were female. Average EF strength was 66.09 ± 17.73 (range 43–93). Average DSMQ-R score was 6.65 ± 2.46 (range 2.17–9.33). The mean duration of interviews was 44 minutes (range 26–88 minutes).

**Table 4.** Characteristics of participants who took part in semi-structured interviews.

Participant	Gender	Age range	Years since diagnosis	Highest level of education	Method of glucose monitoring	Method of insulin delivery
1	Male	51–60	16–25	Post-graduate study	CGM	Self-inject insulin
2	Female	41–50	6–15	Post-graduate study	CGM	Insulin pump
3	Female	41–50	6–15	Post-graduate study	CGM	Insulin pump
4	Male	51–60	26+	Prefer not to say	CGM	Self-inject insulin
5	Female	21–30	1–5	Post-graduate study	CGM	Self-inject insulin
6	Male	61–70	26+	High school/secondary school	CGM	Insulin pump
7	Female	31–40	1–5	College/university	CGM	Self-inject insulin
8	Female	51–60	16–25	Post-graduate study	CGM	Insulin pump
9	Female	41–50	1–5	College/university	CGM	Self-inject insulin
10	Female	51–60	26+	College/university	CGM	Insulin pump
11	Female	51–60	1–5	High school/secondary school	CGM plus and finger pricking	Insulin pump

**Figure 1.**
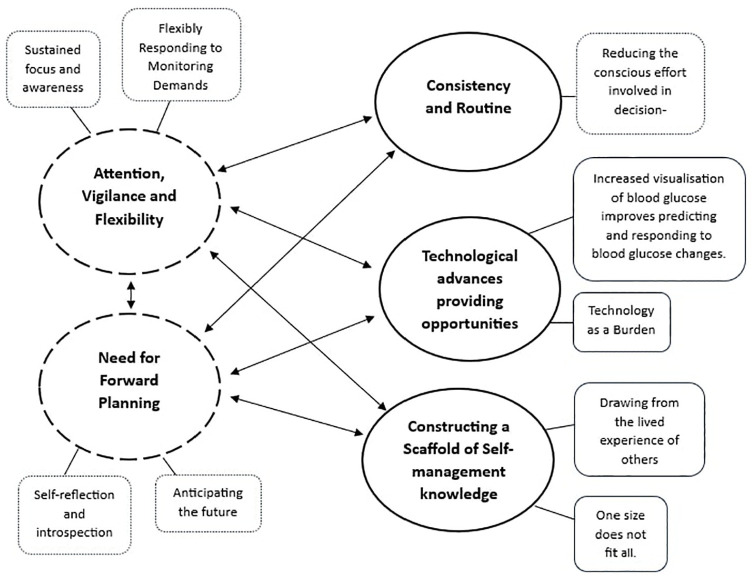
Thematic map showing the relationship between the five main themes relating to perceived difficulties with, and strategies used for self-management, and their subthemes. Dotted lines represent themes associated with self-management difficulties. Solid lines represent themes associated with strategies.

Following thematic analysis, five themes were identified: two relating to perceived difficulties with self-management and three relating to strategies used to aid self-management. These themes are illustrated in Figure 1.

#### Perceived difficulties with self-management

All participants in the study reported difficulties relating to self-management. These fell broadly into two overarching themes: attention, vigilance and flexibility and the need for forward planning.

##### Theme 1: Attention, vigilance and flexibility

A recurring theme was the relentless need to maintain constant attention, vigilance and flexibility around self-management.

P6. ‘*I think the issue is you’ve always got something, you’re always counting something. You’re always looking*’.

Participants described an ongoing demand to remain focussed on their diabetes care. This included maintaining an awareness of activity levels and actively monitoring food consumption such that balance could be created between insulin dose, nutritional content and meal timing.

P3: ‘*Every single thing that goes into your mouth, liquid or food has to be counted, has to be measured*’.

The demand for sustained focus and attention was especially relevant to blood glucose levels, which were universally viewed as random and therefore requiring persistent monitoring and watchfulness.

P10. ‘*it’s a constant looking at your levels, and the thing is even if your levels are perfect, I still check them. I’d never get a day off*’.

The necessity to maintain a flexible approach was further exemplified by one participant who described correcting and recorrecting blood glucose in response to varying levels of daily stress.

P9. ‘*I had a really, really stressful meeting and by the time I got home my levels had skyrocketed. So, then I had to do a correction dose. The problem with that is that when you come out of the anxiety you then drop anyway. So, you then have to catch yourself before you hit too low*’.

##### Theme 2 Need for forward planning

There was a collective view that forward planning was essential for normality in daily life within the limits imposed by self-management demands. This need for planning was felt to reduce spontaneity across a range of daily activities including eating behaviours, family outings, socializing and physical activity.

P2: ‘*I have to be really, really organized in order to live a normal life. Really. And if I’m not, I can’t live a normal life.*

Forward planning involved reflecting on and learning from past experiences and drawing conclusions about the likely impact of a range of confounding variables on future blood glucose levels. This facilitated the prediction of likely blood glucose changes and provided information for future self-management decisions but was perceived as effortful.

P1. ‘*If I want to go into a restaurant, I will need to have a think about where my blood glucose level is at the moment, how much active insulin on board I have from a previous meal, how much exercise I’ve had in the last 6 to 12 hours. You know, whether I’ve been walking around a lot that day or just sitting in front of the telly because again the effect that has on glucose transporters will likely mean I’ll need less insulin*’.

Participants also described a need to anticipate the future such that the occurrence of potential obstacles may be predicted and averted or navigated. This was especially evident in situations where there was unavoidable dependence on the behaviour of others.

P10. ‘*I’m preparing for things going wrong. They may never go wrong but you’ve got to have a plan A and a Plan B*’.

P1. ‘*And then I’ve got to think, well, last time I was in this restaurant, how quickly did they serve me? And have they forgotten my order? You know, things like that. So, I will still try and pre-bolus, but I’m dependent a lot on them actually getting the meal to me before the insulin starts kicking in*’.

#### Strategies used to aid self-management

Several strategies were reported to aid self-management. These clustered into three overarching themes: consistency and routine, technological advances providing opportunities for task delegation and constructing a scaffold of self-management knowledge.

##### Theme 3: Consistency and routine

Seven participants discussed the benefits of consistency and routine, for example describing how consistency relating to food choices reduced the need for effortful calculations when counting carbohydrates and created an automaticity that increased decision-making speed.

*P1. ‘When I look at a meal, I will generally say that’ll be 10 units or 15 units or whatever. And that’s just because I will probably have eaten that type of meal on many occasions before and past experience shows that 10 or 15 units will normally be enough to cover it. It’s just like second nature now. So sure. I mean, I can normally make these decisions within about like 10 seconds’*.

While consistency was described as reducing effort, routines around eating behaviour were also described as leading to a reduction in dietary variety.

P8. ‘*The things that I eat all the time, I’m really good at knowing what I’m doing. We do tend to eat the same, like lots of different salads and different pastas and stuff, but within that range*’.

Routines were evident across all aspects of self-management behaviour with one participant describing aspects of T1D control as becoming habitual and automatic over time.

P5. ‘*You do just get in the habit of doing it. I keep my syringe in a little pencil case. It’s just second nature that I’ll just like pick it up wherever I’m going and then it’s always in my bag. It’s just because you do it every single day, multiple times a day. It’s just, it just becomes there*’.

##### Theme 4: Technological advances provide opportunities for task delegation

Technology was used by all participants to support self-management, most commonly in the form of continuous glucose monitors (CGMs) and insulin pumps. This was felt to transfer a portion of the self-management burden that was experienced, with one participant describing technology as improving their day-to-day living by ‘*orders of magnitude*’ (P1).

All participants had experience of both self-monitoring blood glucose and using CGMs. Most participants reported that CGMs greatly and effortlessly increased the availability of data relating to blood glucose concentration.

P1. ‘*Blood glucose test alone gives me no idea as to whether that’s a stable 5, which is likely to continue, or it’s just passing through 5 on the way to being, you know, too high or too low. Compare that to CGM, I’m getting just shy of 300 readings a day. So, you’re literally talking 50 times more information*’.

Real-time monitoring of blood glucose improved participants’ capacity to predict the direction and speed of blood glucose changes. A range of psychological and emotional benefits were linked to this. Crucially, compared to traditional self-monitoring methods, CGM lessened the burden of identifying patterns of blood glucose change, enabling participants to make informed decisions and respond to potential issues before they happened.

P7: ‘*It gives me a bit of a leg up. You know, if I know I’m going into a meeting in an hour and it bleeps and says your blood’s going to like 4.5, I’ll have something to eat. So, I’m thinking ahead of that time. But that’s all thanks to the technology. It allows me to, you know, be a bit more prepared*’.

However, technology was not always viewed as beneficial. Participants talked about unreliable monitoring data with alarms ‘*going off left right and centre*’ (P11) which caused unnecessary corrections, and data fatigue due to increased workload and the heightened complexity of glucose control. One participant described how insulin pumps increased the burden of decision-making.

P10. ‘*It’s a lot more work because you have more decisions. When you’re on injections, you can inject insulin, you can eat carbs, you can do nothing. That’s it. You have no other options. With a pump. I have those 3. I can also up my basal rate or lower my basal rate. I can change the way I bolus. How am I going to bolus with this food? Am I going to do it all upfront? Am I going to do it over an extended period? Am I going to do a mixture of both? Am I going to split the bolus? Do I need to up my basal rate because I’m running a bit high or is it what I’ve been eating? You’ve suddenly got a lot more options*’.

##### Theme 5: Constructing a scaffold of self-management knowledge

There was a desire among participants to generate a body of knowledge or mental scaffold upon which information could be organized and called upon to enable self-management.

P9. ‘*I’ve got this, and I need to manage it. So, I need to know as much as possible about it*’.

Several sources of knowledge including food packaging, information leaflets, peer-reviewed articles, books and magazines were discussed. Seven participants described the benefit of the lived experience of diabetic peers, with one participant describing peer support as the first port of call for knowledge relating to self-management.

P3. ‘*If I was struggling, I would go to peer support in the first instance*’.

The shared insight of others was applied to improve blood glucose control during physical activity, guide healthy eating and overcome boundaries brought about by diabetes-related complications. One participant discussed how shared experience facilitated decision-making relating to the adoption of an insulin pump.

P10. ‘*She was saying no I thought exactly the same as you, but I do this and oh you can do that. And any questions I brought up, which I now know are the questions everybody asks when they’re going on a pump, what do you do when you sleep or where do you put it. And also, the different strategies you could use for meals like extending your bolus and temporary basil rates. And it just sounded amazing. And I actually walked away from that thinking this could be a really good idea. The real-life experience really tipped it for me*’.

Participants nonetheless noted that unique, individual responses to diabetes meant that the lived experiences of others are not always helpful.

P.11 ‘*I think Doctor Facebook’s worse than Doctor Google because people just pile in with their own experience and try and say that’s gospel, you need to be doing this. But everybody’s different. Fine. Read different things about it but as I’ve discovered with Libre, everybody said, oh, Libre is absolutely wonderful. No, it’s not. It’s not for everybody. Everybody’s different. It affects us all in different ways. So, we can’t possibly all have the same treatment. It’s knowing what works for you*’.

## Discussion

To date, research linking EF to T1D self-management adherence has primarily focussed on children and adolescents (see Ding et al., 2021 for review). This study extends the evidence base by examining the relationship between perceived EF and self-reported self-management in individuals diagnosed with T1D during adulthood. In addition, it examined the associations between specific facets of EF and different self-management tasks and explored whether individuals’ experiences of self-management difficulties and/or use strategies reflect EF processes.

Aligning with previous findings that parent-reported EF deficits predict poor self-management adherence in adolescents with T1D (Perez et al., 2017), this study found that weaker perceived EF also predicted weaker perceived self-management adherence in adults with T1D. In addition, the present results indicate that weaker perceived EF is associated with weaker perceptions of some self-management behaviours (eating, physical activity and glucose monitoring) but not others (medication taking or cooperation with the health care team). All EF sub-facets were correlated with self-management of eating behaviour and physical activity, with no evidence to support a unique association between particular EF sub-facets and these two sets of behaviour. However, only a subset of EF sub-facets (ability to inhibit, self-monitor and plan and organize) were associated with glucose monitoring. None of the specific EF sub-facets correlated with medication taking or cooperation with the healthcare team, suggesting that EF skills are not likely to be key determinants of these behaviours.

Eating behaviours, physical activity and glucose monitoring are all behaviours likely to require EF-related skills such as planning, impulse inhibition and flexible adaptation to change. Both dietary behaviour and physical activity engagement are known to be related to EF strength in the general population (Allan et al., 2016; Daly et al., 2015) as engagement requires the prioritization of long-term benefits (e.g. fitness, health) over short-term costs (e.g. effort, discomfort, inconvenience, resisting temptation from appealing but goal-incongruent options), a balance which requires EF (Hall and Fong, 2007). Similarly, glucose monitoring requires monitoring over time and flexible adaptation of behaviour in response to changes in information (error detection/correction), processes where higher-level executive attention is required (Norman and Shallice, 1986). Although medication non-adherence in chronic conditions has been related to prospective memory impairments (Zogg et al., 2012), insulin adherence was not related to EF in the current sample. This may reflect the fact that medication taking in T1D (insulin) is relatively ‘non-negotiable’, and repeated regularly, making it more habitual and less reliant on cognitive control.

Adherence to medication-taking and healthcare engagement, the other self-management behaviour unrelated to EF in our sample, may also be partially determined by externally controlled, or psychosocial factors which are less dependent on EF. Cooperation with the healthcare team, may be largely outside of the individual’s control, as healthcare contact is typically initiated by healthcare professionals rather than individuals themselves. Consistent with this, a systematic review of 34 studies suggests that diabetic clinic attendance is determined by a complex interplay between multiple factors, including illness perceptions, coping mechanisms and individual relationships with healthcare professionals (Brewster et al., 2020). Logistical issues, such as long waiting times and a lack of clinic flexibility, which are likely beyond the patient’s control, were also reported to contribute to non-attendance. Similarly, a scoping review of 89 qualitative studies concluded that patient-provider communications and the dissemination of knowledge about medication regimes were central to medication adherence in adult patients with various chronic health conditions (Kvarnström et al., 2021). One further possible explanation for the lack of relationship between EF (general or specific) and adherence to medication taking and healthcare engagement is that the sub-sample of participants interviewed in this study reported actively employing strategies designed to reduce cognitive load while performing these behaviours. Regular use of such strategies would be expected to decrease the level of EF required. For example, the use of external cues such as appointment reminders would be expected to reduce the need for internally controlled monitoring and prospective memory in the lead up to appointments. Such strategies have been shown empirically to reduce the number of missed healthcare appointments and increase clinic attendance (Opon et al., 2020).

To better understand the likely contribution of EF to self-management in T1D, qualitative interviews were used in the present study to develop a deeper understanding of participants’ self-management experiences. Based on interview analysis, two core overarching themes around EF-related self-management difficulties emerged: the need for attention, vigilance and flexibility and the need for forward planning. Previous qualitative research concurs that adults newly diagnosed with T1D face disruptions to daily life stemming partly from the constant awareness required for effective diabetes management (Due-Christensen et al., 2019). Similarly, this study found that participants expressed a persistent need to maintain attention, vigilance and flexibility around self-management, particularly concerning blood glucose monitoring and eating behaviour. These activities necessitated frequent checks, calculations and insulin administrations and likely depend on the conscious recruitment of EF which enables activities such as planning, problem-solving and the initiation of appropriate responses to monitoring demands. Participants in this study also described the need to plan for effective self-management which aligns with studies underscoring the importance of planning for chronic illness self-management in general (Audulv, 2013). In the present study, participants described reflecting on past experiences, identifying common patterns of glucose variation, anticipating and planning for future scenarios and appraising potential threats that may hinder self-management in the future. Such pressures likely draw on executive skills including the ability to organize information, analyse situations and evaluate options.

Three themes around strategies for self-management emerged: developing consistency and routine around self-management behaviour, task delegation and the generation of knowledge. Participants identified consistency and routine, for example sticking to known foods, as being strategically useful for reducing the effort, and time required to make self-management decisions. They also reported greater difficulty when eating out of home, where established routines could not be relied upon. This may be explained by the development of automaticity, which partly depends on rehearsal and repetition and reduces the time, effort and conscious awareness required to perform repeated health behaviours (Cummings et al., 2022). Consistent with this, recent studies show that habit formation and perceived behavioural automaticity improve T1D self-management over time (Stone et al., 2023), indicating that a shift away from behavioural control strategies that rely on effortful cognition (EF) is beneficial. This may be particularly relevant for monitoring blood glucose, counting carbohydrates and adjusting insulin doses as perceived automaticity in these domains has been linked to more optimal self-management in adolescents (Cummings et al., 2022). Unlike deliberate, reflective behaviours, habitual or automatic behaviours are considered better predictors of sustained behaviour change (Rothman et al., 2009) and it is recognized that behavioural rehearsal and habit formation are valid techniques to change behaviour (Michie et al., 2013). Therefore, promoting automaticity through the development of consistency and routine could be a valuable strategy for T1D self-management intervention.

Attention, vigilance and flexibility were additionally supported by task delegation, particularly through shifting a portion of the self-management responsibility to technological devices such as CGMs, and insulin pumps. For most participants, CGMs and insulin pumps offered relief from the otherwise relentless demand to pay attention to and monitor aspects of diabetes control. Additionally, they enabled prediction of glucose trends which allowed participants to more easily project into the future and provided an aid to forward planning. Insulin pumps, which operate as an alternative to insulin injections and automatically deliver insulin throughout the day, were associated with improved self-management in the current study. It is likely that insulin pumps not only improve glucose control and reduce the need for frequent manual insulin adjustments but also lessen associated decision-making demands, freeing up time and mental space and allowing individuals to focus more effectively on other EF-driven self-management tasks (e.g. physical activity, meal planning).

Although task delegating was predominantly viewed as beneficial for self-management, it nonetheless came with a price. The increased data made available through CGM paradoxically increased cognitive demand for some participants by expanding the quantity and complexity of information to be considered and self-management decisions to be made. Some participants reported concerns about the reliability of their glucose monitoring devices and experiences of data fatigue. The specific causes of these perceptions were not explicitly stated and fell outside the scope of this study. However, existing research suggests that technology-related burden may arise from multiple factors and can both positively and negatively impact T1D self-management (Abdoli et al., 2017; Montali et al., 2022).

Participants expressed a desire to construct a scaffold of self-management knowledge sourced through written resources and the lived experiences of others. While this strategy helped equip participants with the information necessary to make self-management decisions and aligns with the notion that T1D self-management is facilitated through knowledge and expertise (Abdoli et al., 2017; Adu et al., 2019; Montali et al., 2022), it does not necessarily reduce the demand on executive processes. Indeed, most participants talked about knowledge-based strategies in relation to initial decisions about how to approach or initiate an action rather than in the context of maintaining or self-managing that action over time.

There are several strengths to this study. Notably, this study exceeded the target sample size, increasing the study power, it used high-quality validated measures that included built-in mechanisms to detect problematic or inconsistent responses, and it combined the use of quantitative and qualitative methods to enable triangulation of findings. There are nonetheless limitations. Firstly, it is important to acknowledge the well-documented relationship between depressive symptoms, EF, and T1D self-management. Depression has been linked to both executive dysfunction (McDermott and Ebmeier, 2009) and poorer diabetes self-management (Gonzalez et al., 2008). This raises the possibility that, in this study depressive symptoms may have impacted participants’ ratings of their EF, as well as their self-management behaviours. Depression was not directly measured, and as such, its potential influence on the observed relationships remains unknown. Future research should consider controlling for depressive symptoms to disentangle the unique contributions of EF and mood-related factors to self-management in T1D. Second, the opportunistic recruitment of participants may have introduced sampling bias. Indeed, participants were primarily educated to at least college level thus may not be representative of the broader population of people with T1D. This sample homogeneity could provide one explanation for why education was not a significant predictor of DSMQR score. Third, a key limitation of this study is its reliance on self-report measures, which are subject to recall biases, individual interpretation and potential social desirability effects. While the measures used were validated in previous research and included mechanisms to detect problematic responses, self-report does not provide an objective measure of self-management behaviour. However, the wide range of scores observed did not indicate significant social desirability bias. Future research should aim to incorporate more direct behavioural measures and a measure of resulting glycaemic control to further elucidate the relationship between executive function and self-management in T1D. Finally, this study did not assess participant’s specific method of glucose monitoring in the quantitative study. Given the importance of CGM in diabetes management, future research should explore the impact of different monitoring methods on self-management behaviours.

Future research should focus on understanding the relationship between T1D self-management and performance-based measures of EF which reflect underlying neurocognitive capacity more directly and which are relatively independent of contextual and personality factors that impact an individual’s perception of their EF strength. These performance-based measures could provide a more objective assessment of executive function and help to further clarify the nature of its relationship with self-management behaviours in individuals with T1D.

Previous research has advocated for incorporating cognitive screening into type 2 diabetes self-management care (Cuevas and Stuifbergen, 2017; Primožič et al., 2012). Such screening may help identify individuals who require additional support and enable the customization of interventions to meet their unique needs. Similarly, screening for EF deficits in individuals with T1D may highlight adults at greater risk of facing self-management challenges and thus in greater need of support. Results from the present study indicate that future T1D interventions may benefit from improving self-management automaticity and reducing the cognitive burden associated with self-management tasks.

### Conclusions

To our knowledge, this is the first study to assess the link between EF and self-management in people with an adult diagnosis of T1D. The quantitative results show that (1) EF significantly predicts adherence to T1D self-management, with those with stronger EF reporting better self-management; (2) global EF is associated with the self-management sub-behaviours of eating, glucose monitoring and physical activity, but not with medication taking or cooperation with the healthcare team and; (3) all sub-facets of EF are associated with eating behaviour and physical activity, none are related to medication taking or cooperation with the healthcare team, and only inhibition, self-monitoring and planning are associated with glucose monitoring. Qualitative interviews indicated that individuals with T1D are aware of, and actively work to counteract, EF-related difficulties with self-management including the need for sustained attention, monitoring and flexible responding to demands and planning. Strategies to reduce these demands involved creating consistency and routine and using technology to outsource the cognitive demands of self-management tasks. Further research is required to examine associations between objectively measured EF performance and self-management and to assess the efficacy of reported strategies in improving diabetes management.
